# The Efficacy of MAG-DHA for Correcting AA/DHA Imbalance of Cystic Fibrosis Patients

**DOI:** 10.3390/md16060184

**Published:** 2018-05-26

**Authors:** Caroline Morin, André M. Cantin, Félix-Antoine Vézina, Samuel Fortin

**Affiliations:** 1 SCF Pharma, 235, route du Fleuve Ouest, Ste-Luce, QC G0K 1P0, Canada; cmorin@scfpharma.com; 2 Department of Medicine, Respiratory Division, Faculty of Medicine and Health Sciences, Université de Sherbrooke, Sherbrooke, QC J1H 5N4, Canada; Andre.Cantin@USherbrooke.ca (A.M.C.); Felix-Antoine.Vezina@USherbrooke.ca (F.-A.V.)

**Keywords:** DHA, cystic fibrosis, EFAD, inflammation, medical food

## Abstract

Omega-3 polyunsaturated fatty acid (n-3 PUFA) supplementations are thought to improve essential fatty acid deficiency (EFAD) as well as reduce inflammation in Cystic Fibrosis (CF), but their effectiveness in clinical studies remains unknown. The aim of the study was to determine how the medical food containing docosahexaenoic acid monoglyceride (MAG-DHA) influenced erythrocyte fatty acid profiles and the expression levels of inflammatory circulating mediators. We conducted a randomized, double blind, pilot trial including fifteen outpatients with Cystic Fibrosis, ages 18–48. The patients were divided into 2 groups and received MAG-DHA or a placebo (sunflower oil) for 60 days. Patients took 8 × 625 mg MAG-DHA softgels or 8 × 625 mg placebo softgels every day at bedtime for 60 days. Lipid analyses revealed that MAG-DHA increased docosahexaenoic acid (DHA) levels and decrease arachidonic acid (AA) ratio (AA/DHA) in erythrocytes of CF patients following 1 month of daily supplementation. Data also revealed a reduction in plasma human leukocyte elastase (pHLE) complexes and interleukin-6 (IL-6) expression levels in blood samples of MAG-DHA supplemented CF patients. This pilot study indicates that MAG-DHA supplementation corrects erythrocyte AA/DHA imbalance and may exert anti-inflammatory properties through the reduction of pHLE complexes and IL6 in blood samples of CF patients. Trial registration: Pro-resolving Effect of MAG-DHA in Cystic Fibrosis (PREMDIC), NCT02518672.

## 1. Introduction

A new marine omega-3 compound, a docosahexaenoic acid (DHA) sn1-monoacylglyceride (MAG-DHA), was synthesized in order to evaluate its anti-inflammatory properties in Cystic Fibrosis (CF) patients. This compound showed better absorption of DHA compared with either DHA-triglyceride or DHA-ethyl ester [1]. In addition, its anti-inflammatory effect on the airways has been demonstrated on 2 in vitro models related to CF [2,3,4]. Cystic fibrosis (CF) is the most common fatal autosomal recessive inherited disease in the Caucasian population. CF results from mutations affecting a single gene, located on the long arm of chromosome 7 and encoding for the CF transmembrane conductance regulator (CFTR) protein [5,6]. Malfunction of this chloride channel in CF patients increases viscosity of ductal fluids and is frequently associated with an excessive host inflammatory response. Pancreatic exocrine insufficiency is present even in early infancy in the majority of CF patients, and if untreated, leads to fat malabsorption and malnutrition [7]. Poor nutritional status is a major problem in the vicious cycle of chronic inflammation and its impact in tissue destruction has been well demonstrated [8]. Conditions leading to fat malabsorption, as in CF, have been associated with a high incidence of essential fatty acid deficiency (EFAD). A high EFAD incidence (85%) has been frequently reported in CF patients [9,10,11]. The most often reported abnormalities in CF plasma are a decrease in linoleic acid and docosahexaenoic acid (DHA) as well as an increased in arachidonic acid (AA) [7]. This imbalance in the AA/DHA ratio in favor of AA may contribute to the increase in pulmonary inflammation, resulting in deterioration of the CF patient’s condition. Mechanisms related to EFAD, include the excessive oxidation of EFA as an energy source [12], the exaggerated utilization of eicosanoids as precursors of inflammatory response [13,14], the higher rate of lipid turnover in cell membranes [15], the impaired metabolism of EFA with a defect in plasma membrane incorporation, the decreased activity of desaturases [16] and lipid peroxidation [17]. CF survival and well-being are also correlated with malnutrition. Thus, dietary supplementation with docosahexaenoic acid (DHA) may represent a potentially useful strategy, since DHA is thought to have anti-inflammatory properties. Clinical trials performed on CF patients have revealed that oral supplementation with omega-3 polyunsaturated fatty acid (PUFA) modifies fatty acid profiles in both plasma and cell membrane levels [18]. Moreover, a number of clinical studies have demonstrated improvement of nutritional, clinical, spirometric and inflammatory parameters over time (short and medium term) [19,20,21,22,23]. However, effectiveness of omega-3 PUFA supplementation in cystic fibrosis remains controversial. The aim of this pilot trial was to assess erythrocytes fatty acid profiles, inflammatory parameters (pHLE complexes and IL-6) and lung function following 2 months of daily supplementation of a medical food containing docosahexaenoic acid monoglyceride (MAG-DHA) in CF patients.

## 2. Results

### 2.1. Lipid Profile of Erythrocytes Following MAG-DHA Supplementation

**Figure 1:**
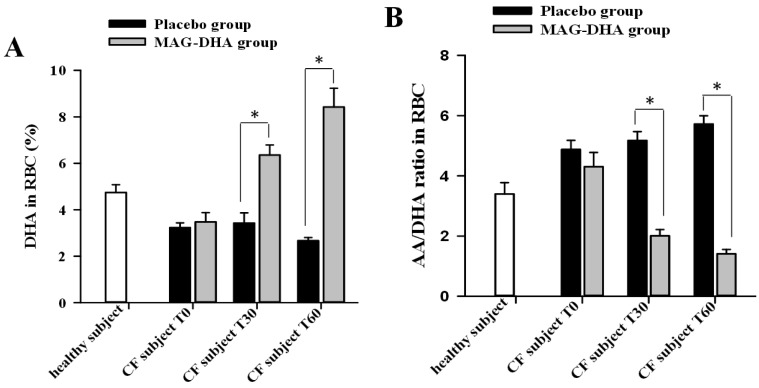
Effect of docosahexaenoic acid sn1-monoacylglyceride (MAG-DHA) supplementation on lipid profile of erythrocytes in Cystic Fibrosis (CF) patients. (**A**) Quantitative analyses of relative DHA levels in erythrocytes (RBC) and (**B**) relative AA/DHA ratio in erythrocytes derived from healthy subjects (*n* = 6), CF patients of placebo group before (T0, *n* = 5) and after supplementation for 30 days (T30, *n* = 5) and 60 days (T60, *n* = 2), as well as from CF patients of MAG-DHA group before (T0, *n* = 6) and after supplementation for 30 days (T30, *n* = 6) and 60 days (T60, *n* = 3), * *p* < 0.05.

Experiments were designed to assess the effect of MAG-DHA intake on the AA/DHA ratio in erythrocytes derived from CF patients. Blood samples were obtained before MAG-DHA or placebo intake (T0) and at 30 (T30) and 60 days (T60) following supplementation. Fatty acid content was thereafter determined by gas chromatography/flame ionization detector (GC/FID) in erythrocytes derived from placebo and MAG-DHA supplemented CF patients. Consistent with previous literature, our results revealed lower DHA levels in red blood cells of CF patients of both groups at T0 (placebo and MAG-DHA) when compared to the levels obtained in healthy subjects (Figure 1A). However, MAG-DHA daily intake by CF individuals significantly increased the DHA levels in red blood cells by 1.9 fold and 3.2 fold following 30 and 60 days of MAG-DHA daily intake, respectively, when compared to the levels obtained from blood samples of the placebo group (Figure 1A). DHA in red blood cells increased from 3.5 ± 0.4% at T0 to 6.4 ± 0.4% at T30 and 8.4 ± 0.8% at T60 for the MAG-DHA group compared to 3.2 ± 0.2% at T0 to 3.4 ± 0.4% at T30 and 2.7 ± 0.1% at T60 for the placebo group. AA level in red blood cells decreased for the MAG-DHA group from 13.9 ± 0.6% at T0 to 12.4 ± 0.7% at T30 and 11.6 ± 0.5% at T60 compared to a stable value for the placebo group 15.4 ± 0.5% at T0 to 15.4 ± 0.5% at T30 and 15.2 ± 0.1% at T60. We also determined the erythrocyte AA/DHA ratio in blood samples of healthy subjects and CF patients by GC/FID (Figure 1B). Lipids analyses revealed that the mean AA/DHA ratios in erythrocytes of CF patients (T0: 4.9 ± 0.3 for placebo and 4.3 ± 0.5 for MAG-DHA group) were higher than the ratios obtained in healthy subjects (3.4 ± 0.3). In contrast, MAG-DHA supplementation of CF patients for 30 and 60 days revealed significant decreases comparatively to the corresponding AA/DHA ratios derived from placebo group. Following MAG-DHA daily intake for 60 days a 3.1 fold decrease in the mean AA/DHA ratio (1.4 ± 0.1) was quantified in CF patients when compared to the baseline ratios found at T0 (4.3 ± 0.5), *p* < 0.05.

### 2.2. pHLE Complexes Levels in CF Patients after MAG-DHA or Placebo Supplementations

**Figure 2:**
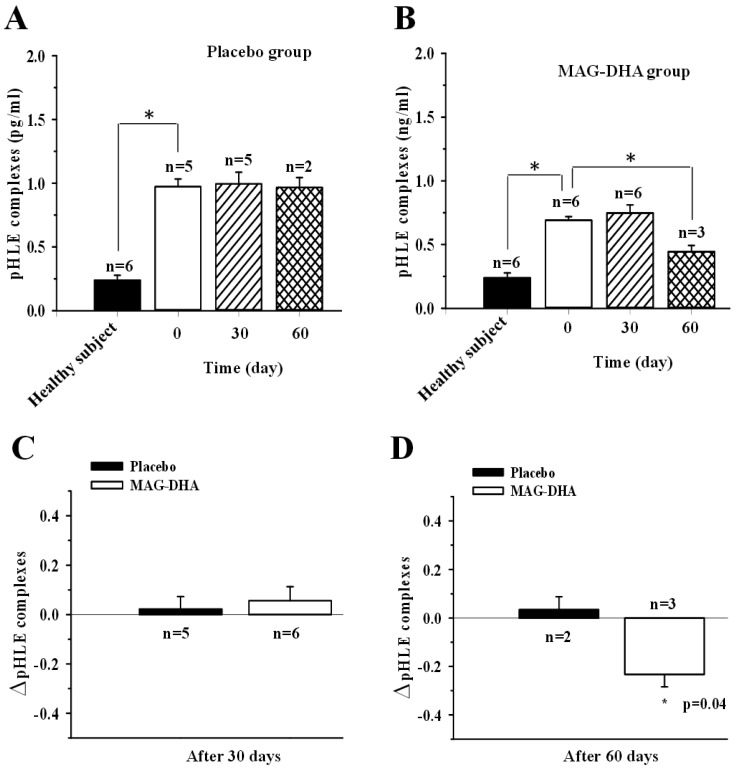
Effect of MAG-DHA on expression levels of alpha1 antitrypsin-deactivated human leukocyte elastase (pHLE) complexes in plasma of CF patients. (**A**) pHLE complex levels were assessed using a specific ELISA in plasma of healthy subjects (*n* = 6) and CF patients of placebo group before (*n* = 5) and after 30 (*n* = 5) and 60 days (*n* = 2) of supplementation. (**B**) pHLE complex levels in plasma of healthy subjects (*n* = 6) and CF patients of MAG-DHA group before (*n* = 6) and after 30 (*n* = 6) and 60 days (*n* = 3) of daily supplementation. (**C**) Bar graph displaying changes in pHLE levels after 30 days of placebo (*n* = 5) or MAG-DHA (*n* = 6) daily intake in CF patients. (**D**) Bar graph displaying changes in pHLE levels after 60 days of placebo (*n* = 2) or MAG-DHA (*n* = 3) supplementation CF patients. Results are expressed as means ± SEM, * *p* ≤ 0.05.

Human leukocyte elastase (HLE) is a toxic neutrophil product, which produces deleterious effects in the CF lung. Active HLE is rapidly inactivated by natural inhibitor, alpha1 antitrypsin (α1AT) and forms a complex (pHLE complexes) that can be detected in the plasma. Several investigators have observed that pHLE complexes are increased in the blood of patients with CF and which is correlated with respiratory exacerbations and lung function [24]. To assess the expression levels of pHLE complexes in plasma of CF patients, specific Enzyme-Linked Immunosorbent Assays (ELISA) were used. Figure 2A,B demonstrated that pHLE complexes were detectable in all healthy volunteers (0.24 ± 0.3 pg/mL). Patients with CF had higher levels of pHLE complexes than did healthy subjects (Figure 2A; 0.97 ± 0.6 for placebo at T0 and 0.69 ± 0.2 pg/mL for MAG-DHA group at T0 versus 0.24 ± 0.3 pg/mL for healthy volunteers, *p* < 0.05). As expected, 30 or 60 days of placebo daily intake did not affect the levels of pHLE complexes in plasma when compared to the corresponding levels at T0 (Figure 2A). Following MAG-DHA daily intake over 60 days, data analyses revealed a significant decrease in pHLE complexes levels compared to the corresponding levels at T0 before supplementation of CF patients (Figure 2B). Figure 2D clearly displays a significant decrease in of pHLE complexes in plasma of CF patients after 60 days of MAG-DHA when compared to the pHLE ratios obtained from the placebo group. However, results revealed that 30 days MAG-DHA supplementation were not able to significantly reduce the pHLE complexes levels in plasma.

### 2.3. Effect of MAG-DHA Intake on Circulating IL-6 Levels in CF Patients

**Figure 3:**
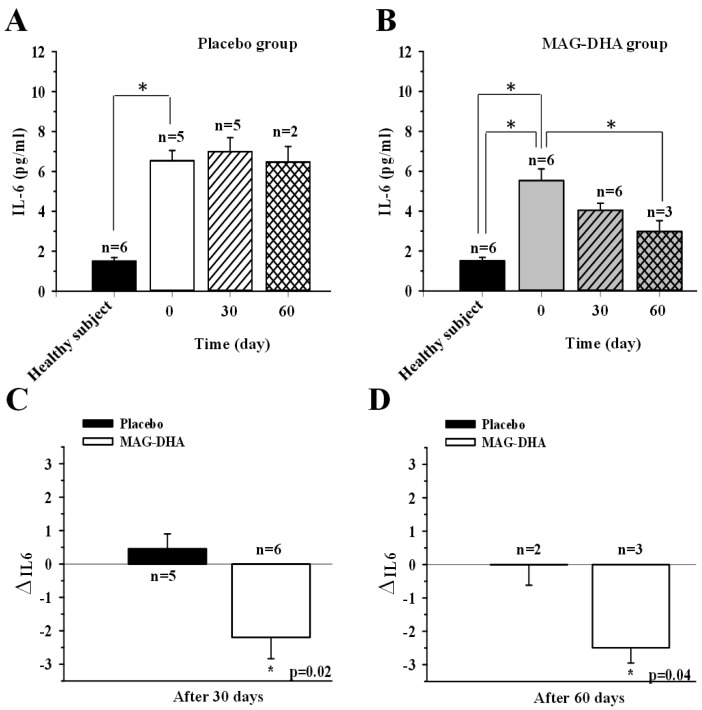
Effect of MAG-DHA on expression levels of circulating IL-6 in CF patients. (**A**) IL-6 levels were assessed using specific ELISA in plasma of healthy subjects (*n* = 6) and CF patients of placebo group before (*n* = 5) and after 30 (*n* = 5) and 60 days (*n* = 2) of supplementation. (**B**) IL-6 levels in plasma of healthy subjects (*n* = 6) and CF patients of MAG-DHA group before (*n* = 6) and after 30 (*n* = 6) and 60 days (*n* = 3) of daily supplementation. (**C**) Bar graph displaying changes in IL-6 levels after 30 days of placebo (*n* = 5) or MAG-DHA (*n* = 6) daily intake in CF patients. (**D**) Bar graph displaying changes in IL-6 levels after 60 days of placebo (*n* = 2) or MAG-DHA (*n* = 3) supplementation CF patients. Results are expressed as means ± SEM, * *p* ≤ 0.05.

Chronic inflammation is a hallmark of CF physiopathology, with elevated levels of various pro-inflammatory cytokines detected in plasma and tissues [25]. Among these, IL-6 is a pivotal component of inflammation initiation and progression [25]. Thus, to evaluate the effect of MAG-DHA, the level of IL-6 was determined by specific ELISA in plasma derived from CF patients and healthy subjects. As expected, the IL-6 level found in plasma derived from CF patients (6.37 ± 1.03 pg/mL for placebo at T0 and 5.11 ± 0.70 pg/mL for MAG-DHA at T0) were increased approximately 4-fold compared to those detected in plasma derived from healthy subjects (1.33 ± 0.17 pg/mL, Figure 3A,B). As expected, 30 or 60 days of placebo supplementation did not change the levels of IL-6 in plasma derived from CF patients when compared to the corresponding IL-6 levels at T0 (Figure 3A). As shown in Figure 3B, MAG-DHA daily intake for 30 and 60 days resulted in a significant decrease in IL-6 levels in plasma of CF patients when compared to corresponding levels at T0 (Figure 3B). Results also demonstrated a significant variation of circulating IL-6 levels of CF patients after 30 days of MAG-DHA daily intake (Figure 3C). Figure 3D clearly displays a significant decrease of IL-6 levels in plasma of CF patients after 60 days of MAG-DHA supplementation when compared to the levels detected in CF patients of placebo group.

## 3. Discussion

### MAG-DHA Increased DHA Bioavailability and Decreased Inflammatory Markers

A precise mechanism to explain alterations in PUFA levels in CF has not been definitively established. A number of hypotheses have been advanced, including increased AA release and eicosanoid metabolism [26], increased flux within PUFA metabolic pathways [27], changes in thiol and phospholipid metabolism [28], as well as increased expression and activity of fatty acid desaturases [29]. Moreover, Njoroge et al. have shown that treatment of CF cells with exogenous DHA reverses the PUFA abnormalities by suppressing expression of Δ5- and Δ6-desaturases [30]. Supplementing omega-3 PUFA could serve to down-regulate the production of inflammatory mediators and thus improve clinical outcomes. Studies have shown that DHA in the form of monoacylglyceride increases the bioavailability of DHA compared to triglycerides [31]. Our previous studies have shown that DHA in monoacylglyceride form displayed an increased bioavailability over free fatty acid in both cells and animal models [1,4,32,33]. In this pilot study, the supplementation was well tolerated by CF patients, despite the omega-3 PUFA supplementation being administered at high doses; MAG-DHA did not modify the body mass index (BMI), platelets and liver enzymes. Our data shows that MAG-DHA is well absorbed and incorporated into erythrocyte membranes after 30 and 60 days of supplementation. A previous clinical study on CF patients by Hanssens, L., et al., [18] have shown a 1.6-fold increase of the DHA erythrocyte level and a 1.2-fold decrease of the AA/DHA ratio after 3 months of supplementation (60 mg/kg omega-3/day with a fish oil triglyceride). Our results show a 1.9-fold increase of the DHA erythrocyte level and a 2.1-fold decrease of the AA/DHA ratio after only 1 month of MAG-DHA supplementation. Moreover, lipid analysis revealed a decreased AA/DHA ratio in erythrocytes that may help decrease lung inflammation in CF subjects. In a previous study we found that MAG-DHA treatment of CF cells decreased AA/DHA ratio and resulted in a decreased expression of mucins, IL-6 and IL-8 [4]. Similarly, CF patients treated with DHA exhibited significant decrease in AA/DHA ratio, both in cell membranes and in plasma phospholipids. DHA supplementation in CF patients, often for short periods of time, was shown to reduce inflammatory markers, although it did not convincingly improve clinical outcomes in most studies [19,20,21,34]. However, a study by Leggieri et al. has shown that six months of DHA supplementation improved the clinical condition and the inflammatory pulmonary and intestinal state of pediatric CF patients [22]. Moreover, Olveira et al. demonstrated that daily omega-3 PUFA supplementation for 12 months in CF patients resulted in an improvement in FEV1, a decrease in exacerbations and course of antibiotics, as well as in inflammatory markers [23]. Thus, the use of the medical food MAG-DHA could represent an improved means to increase daily intake of DHA with a sufficient amount of PUFA to exert anti-inflammatory effects.

Alterations in fatty acids in CF patients may therefore contribute to the inflammatory process leading to progressive tissue damage, and have been correlated with disease severity [7]. Advances in the study of CF lung disease have indicated that pHLE complexes reflect clinically meaningful changes in CF lung disease [24]. In this pilot study, our data suggested that MAG-DHA supplementation for 60 days reduced pHLE complexes and IL-6 levels in blood samples from CF patients. Selective inhibition of these inflammatory markers represents a high-priority target for CF therapy. A study by Yang et al., demonstrated that certain oxidized lipids derived from omega-3 PUFA, such as Resolvins, display a positive relationship with lower inflammatory status and better lung function [35]. The observed reduction of inflammatory markers after 60 days when the AA/DHA ratio was corrected after only 30 days is consistent with the hypothesis of the pro-resolving mediators because mediators must be synthesized from the fatty acids contained in the cell membranes of the target tissues. Therefore enhancing pro-resolving mediators in blood and tissues of CF patients using MAG-DHA, as a stable pharmacological compound, may therefore be of clinical interest. Accordingly, the use of MAG-DHA could be an effective therapeutic approach for increasing the levels of selected pro-resolving lipid mediators, thus reducing the inflammatory state in CF. Despite an improved inflammation profile in 3 CF patients supplemented with MAG-DHA for 60 days, a larger patient population and longer study duration will be required to confirm improvement of pulmonary inflammation and lung function. The major limitation to our study is that it is a pilot study with a small sample size related to the inclusion and exclusion criteria and the study's duration, partly related to the premature termination of the study due to an adverse event not related to the product. Additional studies with larger patient populations and longer study duration are now required to confirm these encouraging results.

## 4. Methods

### 4.1. MAG-DHA

MAG-DHA was obtained by hemisynthesis from a blend of anchovy, sardine and mackerel body oil. Briefly, the blended oil was concentrated to obtain a docosahexaenoic acid rich oil (65% *w*/*w*). The resulting oil was reacted with glycerol and a lipase to obtain a monoglycerides rich oil (MAG-DHA) that complies with the USP monograph of Mono- and Di-glycerides. Certificate of analysis of the clinical batch used in this study can be found as supplementary material. MAG-DHA was given to the patients in the form of oblong 625 mg softgels containing the equivalent of 400 mg of docosahexaenoic acid (DHA).

### 4.2. Clinical Study

Fifteen outpatients with CF, ages 18–48, were recruited from 15 December 2015 to 30 May 2016, at Centre de Recherche Clinique (CRC) du Centre Hospitalier Université de Sherbrooke (CHUS). The study was approved by institutional review boards at CRC CHUS. Inclusion criteria were a diagnosis of CF, age 18 years and older, forced expiratory volume in 1 s (FEV1) between 30–90%, no respiratory exacerbations during the last 2 weeks before the start of the study, no clotting problems or history of bleeding diathesis. Patients with liver function abnormalities were not excluded from the study. Prior to participation, all subjects signed a written informed consent form previously reviewed and discussed with a study physician. Subjects were excluded for the following reasons: pregnancy or women of childbearing potential who were not using a medically accepted means of contraception, and known allergy to fish and/or seafood. Eligible subjects were randomized and divided into 2 groups of 10 patients and received MAG-DHA or sunflower oil as placebo (75% oleic acid, 15% linoleic acid, 4.2% palmitic acid and 3.6% stearic acid) for 60 days. Randomization and treatment assignment were carried out by research pharmacy of CRC CHUS by standard allocation procedures and a fixed block size of 10 subjects per group. Only blind treatment codes were noted on randomization lists provided to study staff. All study staff and participants remained blind to treatment assignment. Patients took 8 × 625 mg MAG-DHA softgels or 8 × 625 mg placebo softgels every day at bedtime. Adherence was determined by softgels count from bottles returned at the next visit. Subjects were evaluated every 30 days for 60 days. The study was terminated prematurely for safety concerns, due to an adverse event in a single patient who developed a marked increase in liver enzymes (>10-fold increase in AST, ALT and bilirubin). Subsequent testing indicated that the participant had contracted viral hepatitis-E confirmed by serology. The hepatitis completely resolved within 3 months. A drug-related adverse event was deemed unlikely. At the time of the premature termination, 11 patients (5 placebo, 6 active drug) had completed 30 days, and 5 had completed 60 days (2 placebo, 3 active drug). Clinical outcome measures at every visit included: AA/DHA ratio in erythrocytes, pHLE complexes and IL-6 levels in plasma as markers of inflammation. Body mass index (BMI) and FEV1/FVC ratio were determined at day 0 before the beginning of the study for placebo and MAG-DHA groups (Table 1).

*Fatty acid composition of erythrocytes using gas chromatography/flame ionization detector (GC/FID):* Fatty acid compositions of erythrocytes derived from CF patients were measured using a modified direct transesterification method in which toluene was used instead of benzene and acetyl chloride was replaced by sulfuric acid [1,2]. Fatty acids were chromatographed as methyl esters on a 60-m fused silica column with an internal diameter of 0.25 mm. Analysis was performed on a Trace GC Ultra gas chromatograph equipped with a flame ionization detector (Thermo Fisher scientific, Waltham, MA, USA).

### 4.3. ELISA Assays

Measurements of inflammatory mediators were determined in plasma by specific ELISA assays for pHLE complexes and IL-6 according to the manufacturer’s instructions (R&D Systems, Minneapolis, MN, USA) [3,22].

### 4.4. Data Analysis and Statistics

Results are expressed as means ± S.E.M. with *n* indicating the number of patients. Statistical analyses were performed using a one-way analysis of variance (ANOVA) and Holm-Sidak as post-hoc test. Differences were considered statistically significant when *p* < 0.05.

## 5. Conclusions

The medical food MAG-DHA represents a simple solution to overcome the impaired absorption of DHA of CF patient. Only 30 days of MAG-DHA supplementation is required to correct erythrocytes AA/DHA imbalance and 60 days for initiating is anti-inflammatory properties through the reduction of pHLE complexes and IL6.

## Figures and Tables

**Table 1:** Study groups characteristics.

Characteristics	Study Groups	
	Placebo (*n* = 5)	MAG-DHA (*n* = 6)
	Mean ± SEM	Mean ± SEM
Age, years	24.4 ± 3.3	32.7 ± 4.6
BMI (kg/m^2^)	20.0 ± 0.5	20.1 ± 0.5
FEV1/FVC	67.7 ± 7.5	63.8 ± 4.3

## Supplementary Materials

The following are available online at http://www.mdpi.com/1660-3397/16/6/184/s1, Certificate of analysis of the clinical batch used in this study.
