# Supplementary material for: The Efficacy of MAG-DHA for Correcting AA/DHA Imbalance of Cystic Fibrosis Patients
Source: Mar Drugs. 2018 May 26;16(6):184. doi: 10.3390/md16060184 (PMC6025526; doi:10.3390/md16060184)
Supplement: Supplementary file 1 [file marinedrugs-16-00184-s001.pdf]

Solutex
Polígono Industrial "El Zafranar",parcela nº 22
50550 MALLEN (Zaragoza) SPAIN
Tel.: +34 918060477 Fax: +34 976 850 123
E-mail: solutex@solutex.es

CERTIFICATE OF ANALYSIS

Product Name: **Solutex0060MG**

Product Description: Eicosapentaenoic & Docosahexaenoic Acid
(OMEGA 3 MONOGLYCERIDE AND DIGLYCERIDE FATTY ACIDS)

Observations: Formulation stabilized with Curcumin and Mixed Natural Tocopherols

Lot: Solutex0060MG0915-0808

Packing Date: 24-Feb-2015 Shelf Life/ Retest Period: 24-Feb-2016

General Description	
Appearance:	Slightly yellow.
Odor:	Slight fish odor.
Solubility:	Almost insoluble in water. Miscible with acetone, ethanol, heptane and methanol.

DETERMINATIONS	SPECIFICATION	RESULT	METHOD
**Fatty Acid Profile**			
DHA mg/g (as FFA)	Min. 600	650.7	Eur.Ph. 2.4.29
Total Omega-3 mg/g (as FFA)*	Min. 600	801.3	Eur.Ph. 2.4.29
Monglyceride content (%A)	Min. 40	40.08	Eur. Ph.2.2.30
*(Sum of 18:3 w3, 18:4 ω 3, 20:4 ω 3, 20:5 ω 3, 21:5 ω 3, 22:5 ω 3, 22:6 ω 3)			
Free Glycerin (mg/g)	Max. 70	9	USP Glycerin RS
**Analytical Data**			
Acid Value (mg KOH/g)	Max. 4	0.28	Eur.Ph. 2.5.1
**Contaminant Data**			
Acetone (mg/kg)	Max. 5000	641	USP <467>
Arsenic (mg/kg)	Max. 0.1	Conforms	AOAC 986.15
Cadmium (mg/kg)	Max. 0.1	Conforms	AOCS Ca18d-01
Lead (mg/kg)	Max. 0.1	Conforms	AOCS Ca18c-91
Mercury (mg/kg)	Max. 0.05	Conforms	AOAC 971.21
Dioxins & Furans (pg WHO-TEQ/g)	Max 1.75	Conforms	EPA 1613
Dioxin- Like PCBs (pg WHO -TEQ/g)	Max. 6.0	Conforms	EPA 1668
PCBs (209 Congeners) (mg/kg)	Max 0.09	Conforms	EPA 8082
Benzo(A)pPyrene (ng/g)	Max. 2	Conforms	Internal Method
**Microbial Contaminants**			
Contaminant fungus (CFU/g)	< 10 000	Conforms	USP 31 <61>
Total Aerobic Count (CFU/g)	< 10 000	Conforms	USP 31 <61>
Escherichia coli (CFU/g)	Absent	Conforms	USP 31 <62>
Salmonella spp. (CFU/g)	Absent	Conforms	USP 31 <62>
Staphylococcus aureus (CFU/g)	Absent	Conforms	USP 31 <62>
Pseudomonas aeruginosa (CFU/g)	Absent	Conforms	USP 31 <62>
**Antioxidant**			
Total curcuminoid content (mg/g)	0.5 – 0.8	0.53	Internal Method
Sum of Tocopherols (mg/g)	0.5 – 0.8	0.60	Internal Method

This product is in accordance with the European legislation and does not contain any GMO.

Storage
Shelf life can be guaranteed if the oil is kept in the original unopened sealed drum, protected from light and heat
If the product is to be manipulated during a long period, it should be preserved each time in an inert atmosphere (nitrogen or argon).
This product is contained in a pre-sealed drum. If strapping is broken at the moment of the inspection, please contact the manufacturer
Standard Packaging Size: 190 Kg

Date: 10- March-2015
QA Approval:

Rev. 1

INSERCO Laboratorios

INFORME SIMPLIFICADO DE RESULTADOS Nº 15-0808-02

CLIENTE: SOLUCIONES EXTRACTIVAS ALIMENTARIAS, S.A.

Nº Reg	15-0808
Ref	SOLUTEX 0060 MG
ID	

PEN	0060
mg muestra pesados	40.62

	EPA	DHA
Relac de Calibración	1.042	1.066
Fecha de calibración	7/25/2014	7/25/2014

		% área cromatog
mg Std int Tricosanoico	6.13	14.15

RESULTADOS

Esteres etílicos		% área cromatog	% de area: % área	% en peso TG: % p/p	% en peso FFA: % p/p
*Ac. Linolénico	18:3 n3		* 0.00	* 0.00	0.00
*Ac. Estearidónico	18:4 n3	0.08	* 0.09	* 0.09	0.08
*Ac. Araquidónico AA	20:4 n6	0.14	* 0.16	* 0.15	0.15
*Ac. EicosaTetrAnoico ETA	20:4 n3	0.2	* 0.23	* 0.22	0.21
Ac. EicosaPentAnoico EPA	20:5 n3	2.74	* 3.19	* 2.90	2.79
*Ac. Eneicosapentaenoico	21:5 n3	0.76	* 0.89	* 0.83	0.80
*Ac. DocosaPentAnoico DPA	22:5 n6	1.73	* 2.02	* 1.88	1.81
*Ac. DocosaPentAnoico DPA	22:5 n3	10.69	* 12.45	* 11.62	11.19
Ac. DocosaHexAnoico DHA	22:6:n3	62.11	* 72.35	67.58	65.07
	suma % area	92.6	* 91.38		
			* Omega 3	83.23	80.13

*I de peroxidos (meqO/Kg)	PEN/0037	
*I de anisidina	PEN/0328	
TOTOX		
*I de peroxidos (meqO/Kg) in situ	PEN/0037	
*I de anisidina in situ	PEN/0328	
*I de acidez (mgKOH/g)	PEN/0036	0.28
*Absorbancia a 233nm	PEN/0054	
*Colesterol (mg/g)	PEN/0288	
*Densidad (g/mL)	PEN/0298	

*Arsénico (mg/Kg)	PEN/0255	
*Plomo (mg/Kg)	PEN/0329	
*Mercurio (mg/Kg)	PEN/0254	
*Cadmio (mg/Kg)	PEN/0329	
*Color Gardner	PEN/0038	
*Agua K-F (mg/Kg)	PEN/0162	
*Glicerol (mg/g)	PEN/0143	9
*Pesticidas	PEN/0250	
Jabon (mg oleato/kg)	PEN/0111	
*PCBs totales (µg/Kg)	PEN/0237	
*Acetona (mg/Kg)	PEN/0232	641

*Oligómeros %A	PEN/0332	<0,1
*Trigliceridos %A	PEN/0332	<0.1
*Digliceridos %A	PEN/0332	6.07
*Monogliceridos %A	PEN/0332	**40.08**
*Gliceridos parciales totales %A	PEN/0332	46.16
*Etil Esteres %A	PEN/0332	53.84

Revisado GDM

La información completa concerniente a los análisis aquí indicados así como la incertidumbre asociada a los mismos se encuentran a disposición del cliente

INSERCO Laboratorios

ANALISIS FISICO QUIMICOS Y MICROBIOLOGICOS
SERVICIOS TECNOLOGICOS

INFORME DE ENSAYO 15/0871-01/01

IDENTIFICACIÓN DEL CLIENTE

NOMBRE SOLUTEX GC, S.L.
DIRECCIÓN Polígono Industrial El Zafranar, Parcela 22
50550 MALLEN
TELÉFONO 976866314 FAX 976850123
CONTACTO

TOMA DE MUESTRAS

Responsable Muestra tomada por el cliente Nº Acta
Fecha de Toma de Muestra Hora
Punto de Toma de Muestra
Procedimiento de Toma de Muestra
Identificación de la Muestra
Contenida en
Observaciones

RECEPCIÓN

MUESTRA DE: Esteres etilicos Nº DE REGISTRO 15/0871-01
S/RFA: SOLUTEX 0060 MG 2
Fecha de Recepción: 24/02/2015 Hora: 18:20
Contenida en: Bote de plástico
Observaciones del Laboratorio

ANÁLISIS

Fecha de Inicio 24/02/2015 Fecha de Fin 09/03/2015

PARÁMETRO	Resultado[1]	P. de Ensayo	Método
Curcumina (mg/g)	0,53	PEN/0094	Cromatografía Líquidos
MixTocoferoles (mg/g)	0,60		Cromatografía de Líquidos

OBSERVACIONES

[1] Los resultados expresados como "<" indican el Límite de Cuantificación del método.
Las incertidumbres de medida se encuentran a disposición del cliente.
Los resultados obtenidos son únicamente representativos de la muestra objeto de los ensayos.
Este documento no podrá reproducirse ni total ni parcialmente, sin la autorización expresa del laboratorio.

09 de marzo de 2015

Gloria Delgado Marquez
Jefe de Laboratorio

LABORATORIOS INSERCO
Avda. Miguel Servet, 81
Parque Empresarial Utebo, nave 18
Tel. 976 78 74 85 - Fax 976 78 74 86
50180 UTEBO (Zaragoza)

Informe emitido por SOLUTEX GC, S.L. propietaria de la marca registrada INSERCO Laboratorios.
Avda Miguel Servet 81 P. Empresarial Utebo nave 18 · Tel. 976787485 - Fax 976787486 · 50180 Utebo (Zaragoza)
E-Mail: inserco@insercolab.com - www.insercolab.com
